# Long-term cardiometabolic health in people born after assisted reproductive technology: a multi-cohort analysis

**DOI:** 10.1093/eurheartj/ehac726

**Published:** 2023-02-06

**Authors:** Ahmed Elhakeem, Amy E Taylor, Hazel M Inskip, Jonathan Y Huang, Toby Mansell, Carina Rodrigues, Federica Asta, Sophia M Blaauwendraad, Siri E Håberg, Jane Halliday, Margreet W Harskamp-van Ginkel, Jian-Rong He, Vincent W V Jaddoe, Sharon Lewis, Gillian M Maher, Yannis Manios, Fergus P McCarthy, Irwin K M Reiss, Franca Rusconi, Theodosia Salika, Muriel Tafflet, Xiu Qiu, Bjørn O Åsvold, David Burgner, Jerry K Y Chan, Luigi Gagliardi, Romy Gaillard, Barbara Heude, Maria C Magnus, George Moschonis, Deirdre Murray, Scott M Nelson, Daniela Porta, Richard Saffery, Henrique Barros, Johan G Eriksson, Tanja G M Vrijkotte, Deborah A Lawlor

**Affiliations:** MRC Integrative Epidemiology Unit at the University of Bristol, Bristol, UK; Population Health Science, Bristol Medical School, University of Bristol, Bristol, UK; MRC Integrative Epidemiology Unit at the University of Bristol, Bristol, UK; Population Health Science, Bristol Medical School, University of Bristol, Bristol, UK; NIHR Bristol Biomedical Research Centre, Bristol, UK; MRC Lifecourse Epidemiology Centre, University of Southampton, Southampton, UK; Singapore Institute for Clinical Science, Agency for Science, Technology, and Research, Singapore, Singapore; Duke-NUS Medical School, Centre for Quantitative Medicine, Singapore, Singapore; Murdoch Children’s Research Institute, Royal Children’s Hospital, Parkville, VIC, Australia; University of Melbourne, Parkville, VIC, Australia; EPIUnit—Instituto de Saúde Pública, Universidade do Porto, Porto, Portugal; Laboratório para a Investigação Integrativa e Translacional em Saúde Populacional (ITR), Porto, Portugal; Department of Epidemiology, Lazio Regional Health Service, Rome, Italy; The Generation R Study Group, Erasmus MC, University Medical Center, Rotterdam, The Netherlands; Department of Paediatrics, Erasmus MC, University Medical Center, Rotterdam, The Netherlands; Centre for Fertility and Health, Norwegian Institute of Public Health, Oslo, Norway; Murdoch Children’s Research Institute, Royal Children’s Hospital, Parkville, VIC, Australia; University of Melbourne, Parkville, VIC, Australia; Department of Public and Occupational Health, Amsterdam Public Health Research Institute, Amsterdam UMC, University of Amsterdam, Amsterdam, The Netherlands; Division of Birth Cohort Study, Guangzhou Women and Children’s Medical Center, Guangzhou Medical University, Guangzhou, China; Department of Paediatrics, Erasmus MC, University Medical Center, Rotterdam, The Netherlands; The Generation R Study Group, Erasmus MC, University Medical Center, Rotterdam, The Netherlands; Murdoch Children’s Research Institute, Royal Children’s Hospital, Parkville, VIC, Australia; University of Melbourne, Parkville, VIC, Australia; School of Public Health, University College Cork, Cork, Ireland; The Irish Centre for Maternal and Child Health Research (INFANT), University College Cork, Cork, Ireland; Department of Nutrition and Dietetics, School of Health Science and Education, Harokopio University, Athens, Greece; Institute of Agri-Food and Life Sciences, Hellenic Mediterranean University Research Centre, Heraklion, Greece; The Irish Centre for Maternal and Child Health Research (INFANT), University College Cork, Cork, Ireland; Department of Obstetrics and Gynaecology, University College Cork, Cork, Ireland; Department of Paediatrics, Erasmus MC, University Medical Center, Rotterdam, The Netherlands; Department of Mother and Child Health, Ospedale Versilia, Viareggio, AUSL Toscana Nord Ovest, Pisa, Italy; MRC Lifecourse Epidemiology Centre, University of Southampton, Southampton, UK; Université Paris Cité and Université Sorbonne Paris Nord, Inserm, INRAE, Center for Research in Epidemiology and StatisticS (CRESS), Paris, France; Division of Birth Cohort Study, Guangzhou Women and Children’s Medical Center, Guangzhou Medical University, Guangzhou, China; K.G. Jebsen Center for Genetic Epidemiology, Department of Public Health and Nursing, Faculty of Medicine and Health Sciences, NTNU, Norwegian University of Science and Technology, Trondheim, Norway; HUNT Research Centre, Department of Public Health and Nursing, Faculty of Medicine and Health Sciences, NTNU, Norwegian University of Science and Technology, Levanger, Norway; Department of Endocrinology, Clinic of Medicine, St. Olavs Hospital, Trondheim University Hospital, Trondheim, Norway; Murdoch Children’s Research Institute, Royal Children’s Hospital, Parkville, VIC, Australia; Department of Paediatrics, University of Melbourne, Parkville, VIC, Australia; Department of Paediatrics, Monash University, Clayton, VIC, Australia; Department of Reproductive Medicine, KK Women’s and Children’s Hospital, Singapore, Singapore; Academic Clinical Program in Obstetrics and Gynaecology, Duke-NUS Medical School, Singapore, Singapore; Department of Mother and Child Health, Ospedale Versilia, Viareggio, AUSL Toscana Nord Ovest, Pisa, Italy; The Generation R Study Group, Erasmus MC, University Medical Center, Rotterdam, The Netherlands; Department of Paediatrics, Erasmus MC, University Medical Center, Rotterdam, The Netherlands; Université Paris Cité and Université Sorbonne Paris Nord, Inserm, INRAE, Center for Research in Epidemiology and StatisticS (CRESS), Paris, France; Centre for Fertility and Health, Norwegian Institute of Public Health, Oslo, Norway; Department of Food, Nutrition and Dietetics, School of Allied Health, Human Services and Sport, College of Science, Health and Engineering, La Trobe University, Melbourne, Australia; The Irish Centre for Maternal and Child Health Research (INFANT), University College Cork, Cork, Ireland; Department of Pediatrics and Child Health, University College Cork, Cork, Ireland; NIHR Bristol Biomedical Research Centre, Bristol, UK; School of Medicine, University of Glasgow, Glasgow, UK; Department of Epidemiology, Lazio Regional Health Service, Rome, Italy; Murdoch Children’s Research Institute, Royal Children’s Hospital, Parkville, VIC, Australia; University of Melbourne, Parkville, VIC, Australia; EPIUnit—Instituto de Saúde Pública, Universidade do Porto, Porto, Portugal; Laboratório para a Investigação Integrativa e Translacional em Saúde Populacional (ITR), Porto, Portugal; Singapore Institute for Clinical Science, Agency for Science, Technology, and Research, Singapore, Singapore; Department of Obstetrics and Gynaecology and Human Potential Translational Research Programme, Yong Loo Lin School of Medicine, National University of Singapore, Singapore, Singapore; Department of General Practice and Primary Health Care, University of Helsinki and Helsinki University Hospital, Helsinki, Finland; Folkhälsan Research Center, Helsinki, Finland; Department of Public and Occupational Health, Amsterdam Public Health Research Institute, Amsterdam UMC, University of Amsterdam, Amsterdam, The Netherlands; MRC Integrative Epidemiology Unit at the University of Bristol, Bristol, UK; Population Health Science, Bristol Medical School, University of Bristol, Bristol, UK; NIHR Bristol Biomedical Research Centre, Bristol, UK

**Keywords:** Blood pressure, Glucose, *In vitro* fertilization, Lipids, Meta-analysis, Pooled longitudinal trajectory analysis

## Abstract

**Aims:**

To examine associations of assisted reproductive technology (ART) conception (vs. natural conception: NC) with offspring cardiometabolic health outcomes and whether these differ with age.

**Methods and results:**

Differences in systolic (SBP) and diastolic blood pressure (DBP), heart rate (HR), lipids, and hyperglycaemic/insulin resistance markers were examined using multiple linear regression models in 14 population-based birth cohorts in Europe, Australia, and Singapore, and results were combined using meta-analysis. Change in cardiometabolic outcomes from 2 to 26 years was examined using trajectory modelling of four cohorts with repeated measures. 35 938 (654 ART) offspring were included in the meta-analysis. Mean age ranged from 13 months to 27.4 years but was <10 years in 11/14 cohorts. Meta-analysis found no statistical difference (ART minus NC) in SBP (−0.53 mmHg; 95% CI:−1.59 to 0.53), DBP (−0.24 mmHg; −0.83 to 0.35), or HR (0.02 beat/min; −0.91 to 0.94). Total cholesterol (2.59%; 0.10–5.07), HDL cholesterol (4.16%; 2.52–5.81), LDL cholesterol (4.95%; 0.47–9.43) were statistically significantly higher in ART-conceived vs. NC offspring. No statistical difference was seen for triglycerides (TG), glucose, insulin, and glycated haemoglobin. Long-term follow-up of 17 244 (244 ART) births identified statistically significant associations between ART and lower predicted SBP/DBP in childhood, and subtle trajectories to higher SBP and TG in young adulthood; however, most differences were not statistically significant.

**Conclusion:**

These findings of small and statistically non-significant differences in offspring cardiometabolic outcomes should reassure people receiving ART. Longer-term follow-up is warranted to investigate changes over adulthood in the risks of hypertension, dyslipidaemia, and preclinical and clinical cardiovascular disease.

## Structured Graphical Abstract

**Structured Graphical Abstract:**
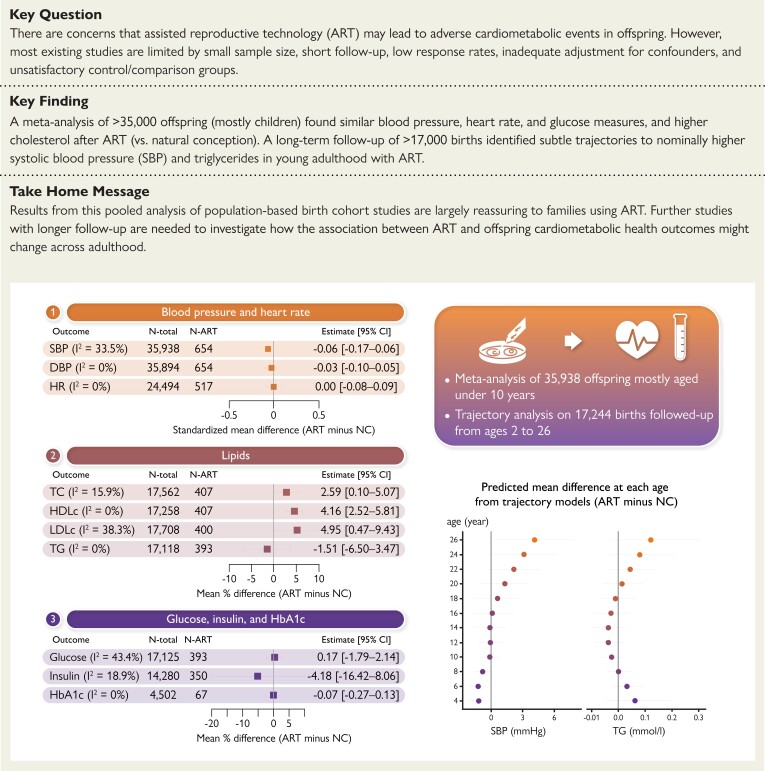
Meta-analysis of results in 35000 offspring with cardiometabolic outcomes measured at various ages found no robust differences in blood pressure, heart rate, triglycerides, or hyperglycaemic/insulin resistance traits, and higher cholesterol in ART-conceived than NC offspring. Analysis of cardiometabolic trajectories up to age 26 years in 17,000 offspring identified subtle increases to nominally higher blood pressure and triglycerides in young adults who were conceived by ART. ART, assisted reproductive technology; NC, natural conception; SBP, systolic blood pressure; DBP, diastolic blood pressure; HR, heart rate; TC, total cholesterol; HDLc, high-density lipoprotein cholesterol; LDLc, low-density lipoprotein cholesterol; TG, triglycerides; HbA1c, glycated haemoglobin.

**See the editorial comment for this article ‘Reassurance for parents with children born via assisted reproductive technology or too soon to tell?’, by C.M. Lawley *et al*., https://doi.org/10.1093/eurheartj/ehac827.**

## Introduction

Use of assisted reproductive technology (ART), which mainly involves *in vitro* fertilization (IVF) or intracytoplasmic sperm injection (ICSI), has risen rapidly in developed countries in recent decades leading to >8 million births worldwide, and this is expected to continue to rise.^1^ There is concern that use of ART may cause adverse cardiovascular and metabolic health outcomes in the offspring.^2–7^ Systematic reviews of mostly small studies report that ART conception is associated with higher offspring blood pressure, glucose, and triglycerides (TG);^5–7^ however, publication and selection bias might influence these findings. Selection bias could arise as most previous studies were clinical cohorts of ART conceptions compared with selected naturally conceived (NC) comparison groups (e.g. family friends) who were not followed up from conception in the same way as those conceived by ART.

A Swiss study published since these reviews that included 54 ART-conceived and 43 NC children discovered signs of premature vascular ageing which persisted at 5-year follow-up assessments at age 17 years, along with new evidence of higher blood pressure that emerged at this older age.^8^ However, family friends were used as NC controls which may introduce a selection bias. A more recent Singaporean population-based birth cohort study where both ART-conceived and NC offspring were selected from the same underlying population and followed up in the same way (*N* = 1178 with 83 ART-conceived offspring) found that ART-conceived offspring had lower blood pressure from age 3 to 6 years.^9^ To the best of our knowledge, no large population-based studies of cardiometabolic health outcomes in ART-conceived offspring, or studies that explored how associations change with increasing age, are available. It is important to explore how associations evolve with age as we cannot assume that associations in early childhood will persist through to adulthood.

Our aim was to conduct a large population-based multicohort study with longitudinal repeated measures analysis to provide more reliable evidence (and so also limiting potential publication bias) on associations between ART conception and long-term offspring cardiometabolic health up to young adulthood. Additionally, we examined the role of underlying parental subfertility,^10,11^ compared associations according to sex^12,13^ and types of ART,^14,15^ and explored if results could be driven by multiple births, preterm birth,^16^ and offspring adiposity.^17^

## Methods

This study was carried out by following a pre-specified analysis plan and code developed by A.E., A.E.T., H.M.I., and D.A.L (https://osf.io/qhwvc/) and is reported in line with the Strengthening the Reporting of Observational Studies in Epidemiology (STROBE) statement.

### Cohort studies

Cohort studies were recruited from the Assisted Reproductive Technology and Future Health (ART-Health) Cohort Collaboration.^18^ ART-Health is a multinational collaboration between 26 cohort studies from the European Union Child Cohort Network, Asia, Australia, and North America.^18–20^ Studies were recruited to ART-Health if they used a population-based study design without selection or oversampling of those conceived by ART, to avoid a selection bias and ensure identical outcome assessment for ART-conceived and NC offspring. Cohort studies were included in the current analysis if they had data on one or more cardiometabolic health measure assessed at any age after birth (in addition to data on whether offspring were conceived by ART or not).

**Figure 1:**
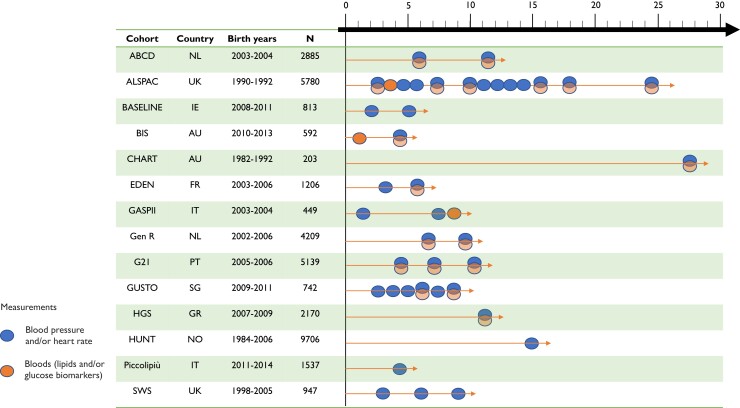
Overview of the included cohorts. The figure shows the birth country, birth years, sample size, and type and age of cardiometabolic outcome assessments in offspring from each included cohort study. The sample sizes represent the maximum number of offspring included in any meta-analysis. ABCD, Amsterdam Born Children and their Development Study; ALSPAC, Avon Longitudinal Study of Parents and Children; BASELINE, Babies After SCOPE: Evaluating the Longitudinal Impact on Neurological and Nutritional Endpoints; BIS, Barwon Infant Study; CHART: Clinical review of the Health of 22–33 years old conceived with and without ART; EDEN, Etude de cohorte généraliste, menée en France sur les Déterminants pré et post natals précoces du développement psychomoteur et de la santé de l’Enfant; GASPII, Gene and Environment: Prospective Study on Infancy in Italy; Gen R, Generation R Study; G21, Generation XXI Study; GUSTO, Growing up in Singapore Towards healthy Outcomes; HGS, Healthy Growth Study; SWS, Southampton Women’s Survey; HUNT, The Trøndelag Health Study. Further details on the included cohorts and measurements can be found in Supplementary material online, *Text S1* and *Table S1*.

In total, 14 of the 26 ART-Health cohorts had these data and were included in this study (*Figure 1*, Supplementary material online, *Text S1*). All offspring with relevant data from each cohort were included in the analysis, without any exclusion criteria such as the exclusion of multiple births or of those with congenital anomalies. Included offspring were born in the UK, Ireland, France, Portugal, Greece, Norway, the Netherlands, Italy, Australia, and Singapore (*Figure 1*). Offspring birth years were from 1982 to 2018, though most were born from 2002 onwards (*Figure 1*). Mean age at cardiometabolic outcome assessment was from 13 months to 27 years, though most cohorts (11/14) had a mean offspring age below 10 years (*Figure 1*, Supplementary material online, *Text S1*).

All included cohorts had approval from their relevant local/national ethics committees and all study participants gave informed consent/assent to participate in the respective cohorts and secondary data analyses. Details on ethics approvals/consent in each cohort can be found in Supplementary material online, *Text S1*.

### Exposure

For our main analysis, a dichotomous variable was derived for each cohort and used to compare offspring conceived using ART with NC offspring. Assisted reproductive technology use in all cohorts was defined in line with the International Glossary on Infertility and Fertility Care definition of ART to cover all interventions that include the *in vitro* handling of both human oocytes and sperm or of embryos for the purpose of reproduction.^21^ This included, but was not limited to, IVF, ICSI, and embryo transfer (ET), though in all cohorts, ART predominantly comprised IVF and ICSI (plus ET). In line with this definition, non-ART methods of medically assisted reproduction (MAR) such as intra-uterine, intra-cervical, or other forms of artificial insemination were excluded from the ART group. Natural conception included those who conceived naturally without any form of MAR.^21^ Assisted reproductive technology conception and NC were identified in each cohort from data on mode of conception and fertility treatment, which were gathered by record linkage or from pregnancy questionnaires (see Supplementary material online, *Text S1*). Given that the use of (any) ART for conception is a major life event and there are legal requirements to provide this information in medical records for many countries, these data are likely to be highly reliable from both record linkage and maternal reports in pregnancy questionnaires.

Where data were available, we further considered whether the ART group were conceived using conventional IVF or ICSI, and whether they were conceived using fresh ET or frozen ET (FET), comparing each subgroup separately to NC. Where data were available, we also considered if the NC group were born to fertile or subfertile parents, depending on the length of time to pregnancy being ≤12 or >12 months since started trying, respectively, comparing each NC subgroup to ART.

### Offspring cardiometabolic outcomes

Eligible offspring cardiometabolic outcomes were systolic blood pressure (SBP, mmHg), diastolic blood pressure (DBP, mmHg), heart rate (HR, b.p.m.), total cholesterol (TC, mmol/L), high-density lipoprotein cholesterol (HDLc, mmol/L), low-density lipoprotein cholesterol (LDLc, mmol/L), TG (mmol/L), glucose (mmol/L), insulin (mU/L), and glycated haemoglobin (HbA1c, %). Similar protocols were followed across all cohorts, and full details on outcome measurements are in Supplementary material online, *Text S1*. Briefly, SBP, DBP, and HR were measured using blood pressure monitors with participants seated and at rest, with SBP, DBP, and HR calculated as the average of first, second, and (if available) third measurements. Biomarkers (lipids and hyperglycaemic/insulin resistance markers) were obtained using standard clinical laboratory procedures in fasting or non-fasting blood samples, depending on age.

To maximize sample size for the meta-analysis (of results from all cohorts), where a cohort had repeated measurements of an outcome (i.e. measures taken at different follow-up timepoints/waves/mean ages), we selected the age of the outcome measure that had the largest number of offspring for meta-analysis (see Supplementary material online, *Table S1*). Additionally, associations of ART with trajectories of change in cardiometabolic outcomes included all repeat measurements from individual cohorts where these were available for sharing.

### Confounders

We used a Directed Acyclic Graph (see Supplementary material online, *Figure S1*), developed with input from the multidisciplinary author group, to identify (and control) for confounders and avoid over-adjustment for mediators.^22–24^ Priority was given to confounders that were available in most of the included cohorts as well as for most of the cohort offspring. This identified the following potential confounders: maternal age at pregnancy/birth, parity, pre-pregnancy body mass index (BMI) and smoking, education (as a marker of socioeconomic position) and ethnicity. Most cohorts (*n* = 12) had data on all confounders; two cohorts (HUNT and CHART) were unable to adjust for maternal BMI, smoking, or ethnicity (though for HUNT, 97% of the population had European ancestry), with one cohort (HUNT) also unable to adjust for maternal education. Details on confounder measurement are in Supplementary material online, *Text S1*.

### Statistical analysis

#### Analysis of all cohorts (various ages)

Associations of ART conception with cardiometabolic outcomes were examined separately in each cohort and results were subsequently combined through meta-analysis. Cohort-specific multivariable linear regression models were used to estimate mean difference and 95% confidence intervals (CIs) in cardiometabolic outcomes between ART-conceived and NC offspring. Models were adjusted for confounders (maternal age, education, parity, BMI, smoking, and ethnicity) plus offspring sex and exact age at outcome assessment (sex and age were included to control for variation in outcomes related to sex and age and improve precision of estimates). Robust standard errors were used by cohorts that had any related individuals. This approach was chosen due to the low prevalence of relatedness within the cohorts, for example, the overall proportions of multiple births (twins, triplets, or higher order births) in the six cohorts that included multiple births ranged from 1.1% to 5.0% (see Supplementary material online, *Table S2*).

To facilitate comparison of effect sizes between SBP, DBP, and HR, these were presented as standardized regression coefficients, after standardizing to cohort-specific standard deviation (SD) units (mean = 0 and SD = 1). To aid interpretability of results, SBP, DBP, and HR were also analysed in their original units. Because height is strongly related to blood pressure in childhood,^25^ SBP and DBP were additionally analysed as percentile ranks after age-, sex- and height-standardization informed by guidelines from the National Heart, Lung, and Blood Institute (NHLBI) and Centers for Disease Control and Prevention (CDC).^26^ This analysis was only done in cohorts that had blood pressure measured before age 18 years, because the NHLBI/CDC guidelines only provide percentiles up to age 17 years as the association with height is less prominent in adulthood. Lipids, glucose, and insulin were analysed after natural log transformation, and the results were presented as percentage (%) differences between ART-conceived and NC offspring.^27^ This was done because these biomarkers, and hence the regression model residuals, were right skewed and to facilitate comparability of results across markers. HbA1c was analysed in its original units (i.e. % of glycosylation).

Cohort results were pooled using a random-effects meta-analysis (DerSimonian and Laird estimator with the Hartung–Knapp–Sidik–Jonkman variance adjustment) to incorporate between-cohort heterogeneity, including between-cohort differences in offspring birth years and country of birth, and obtain mean differences (and 95% CIs) in outcomes across all cohorts. The *I*^2^ statistic was used to quantify the consistency in the pooled estimates as the percentage of total variability due to between-cohort heterogeneity.^28^ The robustness of the pooled results to influential cohorts was investigated by using a leave-one-out sensitivity analysis where the meta-analysis was repeated by leaving one of the cohorts out each time.^29^ The contribution of each cohort to overall heterogeneity and its impact on the pooled estimates were graphically represented in a modified Baujat plot.^29^

The following pre-specified subgroup analyses were performed to further explore sources of heterogeneity. We attempted to separate out effects of ART conception from effects of parental subfertility by repeating analyses comparing ART-conceived with NC offspring from subfertile parents and to offspring from fertile parents. Differences by sex and ART treatment were explored by repeating analyses stratified by sex; comparing IVF and ICSI separately to NC; and comparing fresh ET and FET separately to NC and inspecting the difference in effect sizes between groups. Differences between subgroups were examined by a Wald test.^30,31^ Lastly, we explored whether results were driven by twins/multiple births by repeating analyses in singletons and examined if results were driven by preterm birth and offspring adiposity by refitting the main (confounder-adjusted) models with extra adjustment for offspring birth weight, gestational age at delivery, and BMI (before or at outcome assessment).

#### Age-change (2–26 years) trajectory analysis

Differences in cardiovascular (SBP, DBP, HR), lipids (TC, HDLc, LDLc, TG), and glucose trajectories from childhood to young adulthood between ART-conceived and NC offspring were examined in four cohort studies that all collected repeated measurements: (i) the UK-based Avon Longitudinal Study of Parents and Children (ALSPAC) with 1–12 repeated blood pressure and HR measures, 1–7 repeated lipids measurements (from age 2 to 26 years), and 1–4 repeated glucose measures from age 7 to 26 years,^32–34^ (ii) the Portuguese G21 cohort with 1–3 repeated measurements for all outcomes at ages 4, 7, and 10 years,^35^ (iii) the Amsterdam Born Children and their Development study (ABCD) with up to 2 repeated measures for all outcomes at ages 5 and 11 years,^36^ and (iv) the Growing up in Singapore Towards healthy Outcomes (GUSTO) study^37^ with 1–6 repeated SBP, DBP, and HR measurements from age 3 to 8 years, and 2 lipids and glucose measurements at ages 6 and 8 years.

Associations of ART conception with mean cardiometabolic health trajectories were examined using natural cubic spline mixed-effects models.^38^ Mean cardiometabolic health trajectories were modelled using a natural cubic spline function for age (as a fixed effect) to allow for nonlinear change in outcomes with age. The complexity of the trajectory shape was selected by comparing models with different numbers of knots placed at quantiles of the age distribution, and selecting models based on combination of fit indices, biologically plausible fitted trajectory,^12^ and avoidance of overfitting by choosing models with a fewer number of knots.^38^ The selected models included three (SBP, DBP, and HR), two (TC, HDLc, LDLc, TG), and one (glucose) knot(s) in the natural cubic spline function. Models were adjusted for sex and confounders and included an adjustment for cohort (as a fixed effect) to control for between-cohort differences. An interaction term between ART and age was included in all models to allow different mean trajectories for ART-conceived and NC groups.^38,39^ All models included random intercept and random linear slope for age to allow for between-individual differences at baseline and in change with age. Predicted mean trajectories and differences between ART and NC were calculated.

We explored whether differences in mean trajectory were driven by multiple births by repeating models in singletons only and whether differences were driven by preterm birth and offspring adiposity by refitting the main confounder-adjusted models with extra adjustment for birth weight, gestational age, and offspring sex-specific BMI-for-age *Z*-score (taken before the first outcome measurement and standardized to the WHO Growth reference standards). Lastly, differences in trajectories of SBP and DBP percentiles up to age 18 years (after age-, sex-, and height-standardization to NHLBI/CDC guidelines) were examined. Analysis was done in R version 4.0.2 (R Project for Statistical Computing).^40–42^

#### Missing data

For the analyses of all cohorts (with various ages), all offspring were included if they had complete data on mode of conception, confounders, and the cardiometabolic outcome of interest. For the age-change trajectory analyses, all offspring were included provided they had complete data on mode of conception and all confounders, plus data for one or more of the specified repeated outcome measurements. Therefore, selection bias due to missing outcome data may be reduced in the trajectory models by including all offspring with incomplete outcome values, with estimation by maximum likelihood, under the missing at random assumption (i.e. the probability that an outcome value is missing depends on observed values of the outcome, conditional on the covariates in the model). To explore the potential impact of missing data, we compared characteristics between included offspring and those that were excluded due to missing data (see Supplementary material online, *Table S2*).

## Results

### Participant characteristics

A total of 14 cohorts were included (two cohorts each from the UK, the Netherlands, Italy, and Australia, and one cohort each from Ireland, France, Portugal, Greece, Norway, and Singapore). The number of cohorts and offspring included in the main meta-analysis (i.e. ART compared with NC) ranged from 14 cohorts and 35 938 (654 ART) offspring for SBP to 2 cohorts and 4502 (67 ART) offspring for HbA1c. The number of cohort offspring in each analysis and the mean and SD of outcomes and ages at each outcome assessment are given in Supplementary material online, *Table S1*. Those excluded due to missing data had lower maternal education and higher prevalence of pregnancy smoking but were broadly similar on the other maternal factors (see Supplementary material online, *Table S2*). Compared with NC, offspring conceived using ART had a lower birth weight and gestational age, higher prevalence of multiple births, and they were more likely to have nulliparous mothers, mothers with older age at pregnancy, mothers who were more educated, and mothers who were less likely to have smoked in pre-/early pregnancy (see Supplementary material online, *Table S3*).

### Results of analysis with all cohorts/ages

**Figure 2:**
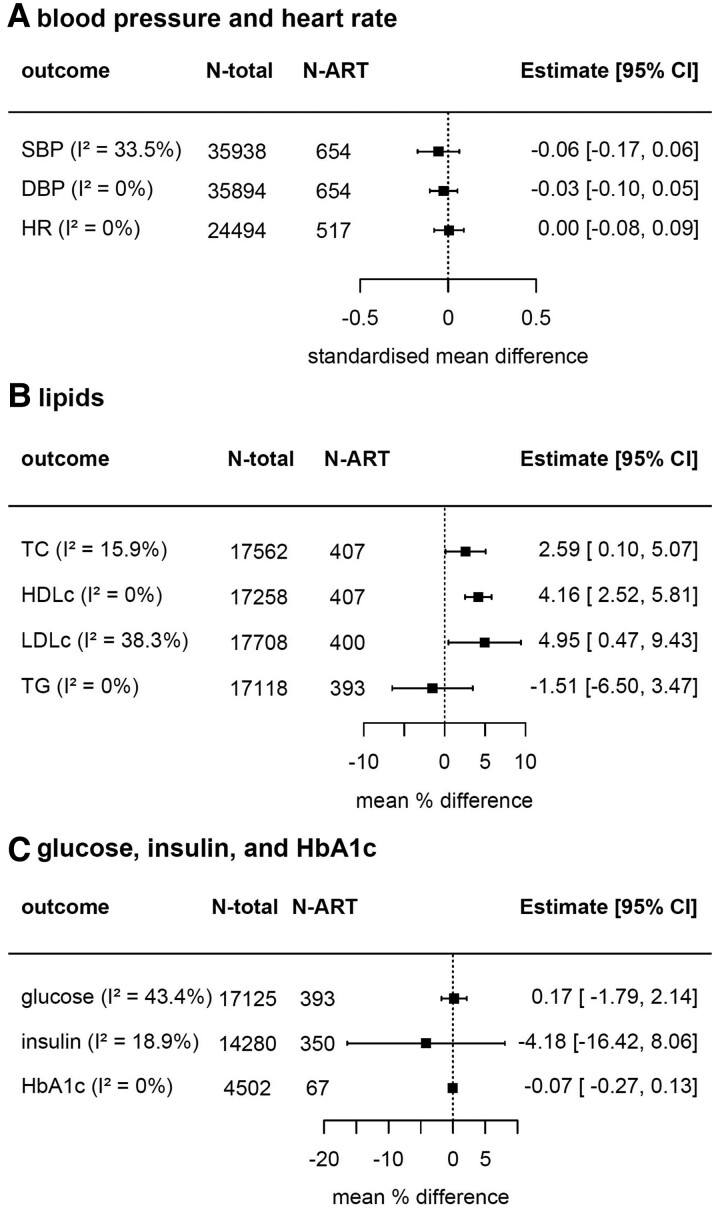
Pooled mean differences in cardiometabolic health outcomes between assisted reproductive technology-conceived and natural conception offspring from up to 14 birth cohort studies. The figure shows the pooled confounder-adjusted mean differences (and 95% confidence intervals) in cardiometabolic outcomes between assisted reproductive technology-conceived and natural conception offspring from up to 14 cohort studies. Estimates represent standardized mean differences in (A) systolic blood pressure, diastolic blood pressure, and heart rate, (B) mean % difference in lipids, (C) glucose, and insulin, and mean difference in % glycosylation for HbA1c. Cohort-specific models were adjusted (as fully as possible) for maternal age, parity, education, smoking, body mass index, and ethnicity, plus offspring sex and age at outcome assessment. Results from each cohort are presented in Supplementary material online, *Figure S2*. ART, assisted reproductive technology; NC, natural conception; SBP, systolic blood pressure; DBP, diastolic blood pressure; HR, heart rate; TC, total cholesterol; HDLc, high-density lipoprotein cholesterol; LDLc, low-density lipoprotein cholesterol; TG, triglycerides; HbA1c, glycated haemoglobin. The pooled mean differences in blood pressure and heart rate in original units were −0.53 mmHg (−1.59 to 0.53) for systolic blood pressure, −0.24 mmHg (−0.83 to 0.35) for diastolic blood pressure, and 0.02 b.p.m. (−0.91 to 0.94) for heart rate.

The pooled confounder-adjusted mean differences in each cardiometabolic outcome between ART-conceived and NC offspring are presented in *Figure 2*, with results from each individual cohort presented, arranged by mean age, in Supplementary material online, *Figure S2*. There were no statistically significant pooled differences (at the conventional *P* < 0.05 threshold) in SBP (standardized mean difference between ART-conceived and NC groups across all cohorts: −0.06 SD; 95% CI: −0.17 to 0.06), DBP (−0.03 SD; −0.10 to 0.05), and HR (0.00 SD; −0.08 to 0.09). The equivalent mean differences expressed in original units were −0.53 mmHg (−1.59 to 0.53) for SBP, −0.24 mmHg (−0.83 to 0.35) for DBP, and 0.02 b.p.m. (−0.91 to 0.94) for HR. Mean TC (mean % differences: 2.59%; 0.10–5.07), HDLc (4.16%; 2.52–5.81), and LDLc (4.95%; 0.47–9.43) were all significantly higher in ART-conceived than NC offspring. No significant differences were found in mean TG (−1.51%; −6.50 to 3.47), glucose (0.17%; −1.79 to 2.14), insulin (−4.18%; −16.42 to 8.06), or HbA1c (−0.07% glycosylation; −0.27 to 0.13).

There was no observed heterogeneity (*I*^2^ = 0%) between cohorts for DBP, HR, HDLc, TG, and HbA1c (*Figure 2*, Supplementary material online, *Figure S3*). Between-cohort heterogeneity was unlikely to be important for TC (*I*^2^ = 15.9%) and insulin (*I^2^* = 18.9%), and there was moderate between-cohort heterogeneity for SBP (*I*^2^ = 33.5%), LDLc (*I*^2^ = 38.3%), and glucose (*I*^2^ = 43.4%) results (*Figure 2*, Supplementary material online, *Figure S3*). Sensitivity analyses showed that GUSTO had both the largest relative contribution to heterogeneity and impact on the pooled SBP results (see Supplementary material online, *Figure S3*), with the pooled estimate slightly attenuated when GUSTO was removed (standardized mean difference in SBP across all cohorts after GUSTO is excluded: −0.02 SD; −0.13 to 0.09, *I*^2^ = 12.8%). The pooled estimate for LDLc was influenced by the G21 and Gen R cohorts (see Supplementary material online, *Figure S3*); removing G21 pulled results to higher LDLc with ART (6.42%; 2.42–10.43, *I*^2^ = 0%), whereas removing Gen R attenuated the difference (3.54%; −0.67 to 7.73, *I*^2^ = 20.0%). No clearly influential cohorts were identified for glucose result (see Supplementary material online, *Figure S3*).

**Figure 3:**
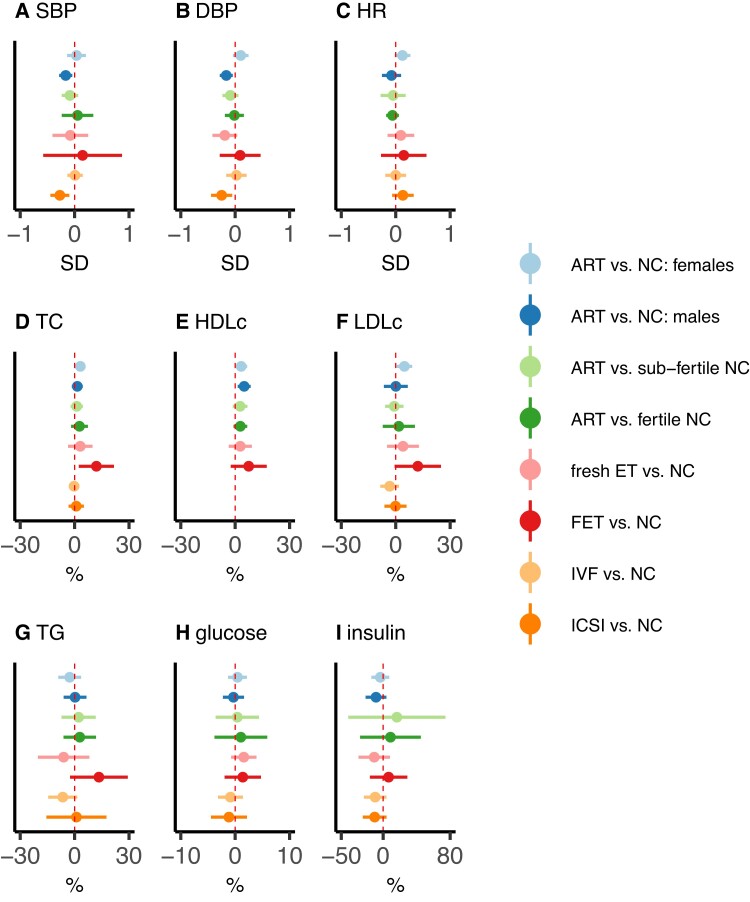
Pooled mean differences in cardiometabolic health outcomes between assisted reproductive technology-conceived and natural conception offspring, stratified by sex, parental subfertility, fresh embryo transfer/frozen embryo transfer, and *in vitro* fertilization/intracytoplasmic sperm injection. The figure shows pooled confounder-adjusted mean differences in cardio-metabolic outcomes between pre-specified assisted reproductive technology-conceived and natural conception offspring subgroups. Estimates represent standardized mean differences in (A) systolic blood pressure (SBP), (B) diastolic blood pressure (DBP), and (C) heart rate (HR), and mean % difference in (D) total cholesterol (TC), (E) high-density lipoprotein cholesterol (HDLc), (F) low-density lipoprotein cholesterol (LDLc), (G) triglycerides (TG), (H) glucose, and (I) insulin. The horizontal bars represent 95% confidence intervals. Cohort-specific models were adjusted (as fully as possible) for maternal age, parity, education, smoking, body mass index, and ethnicity, plus offspring sex and age at outcome assessment. ART, assisted reproductive technology; NC, natural conception; IVF, *in vitro* fertilization; ICSI, intracytoplasmic sperm injection; ET, embryo transfer; FET, frozen embryo transfer. The corresponding numerical results, and results from tests of subgroup differences, are given in Supplementary material online, *Table S4*.

*Figure 3* presents results for all pre-planned subgroup analyses, with all numerical results, including results from tests of differences between subgroups, in Supplementary material online, *Table S4*. All results were consistent between those with and without parental subfertility, and for most results comparing females to males, fresh ET to FET, and conventional IVF to ICSI. Associations of ART conception (vs. NC) with SBP and DBP were stronger in males compared with females, and in offspring conceived using ICSI compared with IVF (vs. NC). Associations of ART conception (vs. NC) with HR were stronger in females compared with males, and associations with TG were stronger for FET vs. NC compared with fresh ET. The *P*-values from tests of between subgroup differences for all of these were <0.1, but differences tended to be small. For example, the standardized mean differences (ART minus NC) in SBP were −0.16 SD (−0.28 to −0.04) in males and 0.03 SD (−0.14 to 0.21) in females.

Results in singleton births (see Supplementary material online, *Figure S4*) were consistent with results in all participants (i.e. with both singletons and multiple births included). Differences in SBP and SBP were slightly increased after extra adjustment for birth weight, gestational age, and offspring BMI, and results (with the extra adjustments) were consistent with the confounder-adjusted results for all other outcomes (see Supplementary material online, *Figure S5*). Lastly, analyses on age-, sex-, and height-standardized blood pressure percentiles (in 13 cohorts with measures before age 18 years) were consistent with the main results, with no significant pooled differences in percentile rank for SBP (−0.01; −0.03 to 0.02, *I*^2^ = 32.9%) or DBP (0.00; −0.01 to 0.01, *I*^2^ = 0%).

### Results of age-change trajectory analysis

**Figure 4:**
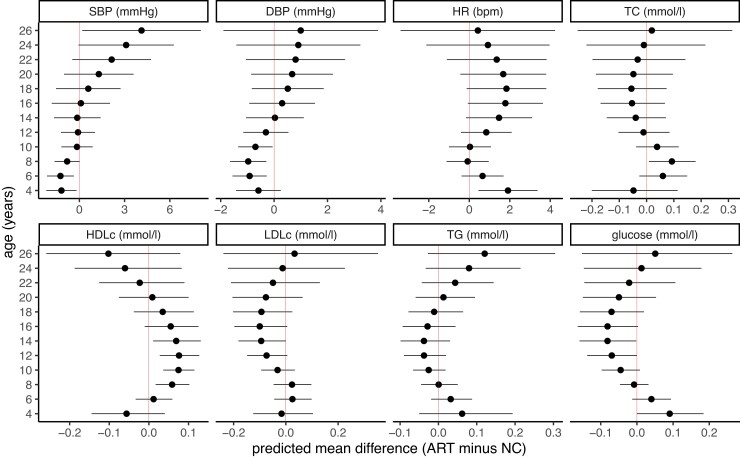
Predicted mean differences in cardio-metabolic trajectories from childhood to adulthood between assisted reproductive technology-conceived and natural conception offspring. The figure shows the predicted mean differences in cardio-metabolic outcomes from childhood to adulthood between the assisted reproductive technology-conceived and natural conception offspring. The horizontal bars represent 95% confidence intervals. Predicted means were obtained from multicohort (ABCD, ALSPAC, G21, and GUSTO) natural cubic spline mixed-effects models that were adjusted for offspring sex, maternal age, parity, body mass index, smoking, education, ethnicity, and cohort. All models included an interaction between assisted reproductive technology and age. SBP, systolic blood pressure; DBP, diastolic blood pressure; HR, heart rate; TC, total cholesterol; HDLc, high-density lipoprotein cholesterol; LDLc, low-density lipoprotein cholesterol; TG, triglycerides. The corresponding numerical results are given in Supplementary material online, *Table S5*. The predicted mean trajectories are presented in Supplementary material online, *Figure S6*.

A total of 17 244 (244 ART), 16 818 (243 ART), 139 126 (188 ART), and 13 386 (184 ART) offspring were included in pooled trajectory analysis for blood pressure, HR, lipids, and glucose, respectively. The age range of outcome assessment was from 2.9 to 26.5 years. The predicted mean cardiometabolic health trajectories for ART-conceived and NC offspring are presented in Supplementary material online, *Figure S6*, and the predicted mean differences in each outcome from childhood to adulthood are presented in *Figure 4* and Supplementary material online, *Table S5*.

Blood pressure was lower during childhood in ART-conceived offspring [e.g. the predicted differences (ART minus NC) at age 6 years were −1.26 mmHg (−2.15 to −0.37) for SBP and −0.92 mmHg (−1.56 to −0.28) for DBP]. Blood pressure was similar in both groups during adolescence, with some evidence of a trend towards higher blood pressure (SBP) in young adulthood in ART-conceived offspring: the predicted difference at age 26 years was 4.12 mmHg (0.19–8.06) for SBP and 1.00 mmHg (−1.90 to 3.89) for DBP. Heart rate was mostly slightly higher for ART-conceived offspring throughout the follow-up, but the difference was small, e.g. predicted differences at ages 4 and 26 years were 1.91 b.p.m. (0.45–3.37) and 0.42 b.p.m. (−3.39 to 4.24), respectively.

There was no evidence of difference in TC at most ages except for higher levels at age 8 years in ART-conceived offspring [predicted difference in TC: 0.09 mmol/L (0.01–0.18)]. High-density lipoprotein cholesterol was higher in ART-conceived offspring from childhood to adolescence and there was a trend towards lower HDLc in young adulthood, e.g. predicted differences in HDLc at age 14 and 26 years were 0.07 mmol/L (0.01–0.13) and −0.10 mmol/L (−0.26 to 0.08), respectively. TG were broadly similar during childhood and adolescence with some evidence of a trend to higher TG in young adulthood with ART, but the difference was small: predicted differences in TG at age 26 years was 0.12 mmol/L (−0.03 to 0.30). No noticeable differences were seen in LDLc or glucose trajectories (*Figure 4*).

The predicted mean trajectories from analyses in singletons (see Supplementary material online, *Figure S7*), and after extra adjustment for offspring birth weight and gestational age (see Supplementary material online, *Figure S8*), and childhood BMI (see Supplementary material online, *Figure S9*) were consistent with results from the main confounder-adjusted models. Lastly, analyses on age-, sex-, and height-standardized blood pressure percentile trajectories (up to age 17 years) were consistent with the trajectories to this age from the main blood pressure trajectory analysis (see Supplementary material online, *Figure S10*).

## Discussion

To the best of our knowledge, this is the largest study focusing on cardiovascular and metabolic outcomes in ART-conceived offspring, and the longest follow-up of its kind. Our meta-analysis of results from >35 000 offspring combining outcomes measured at any age (although including mostly children aged <10 years) found no robust differences in blood pressure, HR, TG, or hyperglycaemic/insulin resistance traits, and a higher cholesterol in ART-conceived offspring. Complementary analyses on cardiometabolic health trajectories from ages 2 to 26 years in >17 000 offspring identified a lower blood pressure during childhood and subtle increase, resulting in higher blood pressure and more atherogenic dyslipidaemia (higher TG and lower HDLc) in young adults who were conceived by ART (*Structured Graphical Abstract*). Results were similar in males and females, when comparing ART-conceived offspring with NC offspring with/without parental subfertility, conventional IVF and ICSI with NC, fresh ET and FET with NC, and when restricted to singletons, and after extra adjustment for offspring birth weight, gestational age, and BMI.

The lower blood pressure trajectory up to age 8 years with ART (and the modestly lower SBP but not reaching *P* < 0.05 across all cohort) is consistent with results from a Singaporean birth cohort showing lower SBP in ART-conceived offspring across four timepoints from age 3 to 6 years.^9^ Our results suggest that this trajectory might subsequently change to higher blood pressure from older adolescence in those conceived by ART, which supports and adds to findings from a small Swiss clinical ART cohort showing that higher blood pressure in offspring conceived via ART was only observed at age 17 years, despite evidence of premature vascular ageing found at baseline (mean 12 years) and 17 years.^8^ We found no overall difference in HR or evidence of emerging/long-term associations with HR, which supports and expands on results from the only previous study (to our knowledge) of this association in 9-year-olds from a small clinical ART cohort in the Netherlands.^43^

Our findings suggest that TG were similar but that TC (HDLc and LDLc) was higher (across all cohorts) in the ART group in childhood. Trajectory analyses suggested that the higher TC and LDLc levels were largely restricted to childhood with no differences by young adulthood. In contrast, with older age, TG increased and HDLc decreased in those conceived by ART compared with NC, such that by age 26 years TG were higher, and HDLc lower, in those conceived by ART, though differences were small and not statistically significant. Only few studies, done on mostly young children, examined associations with lipids, with these showing inconsistent results of no difference or lower TG and LDLc, and no difference or higher HDLc with ART conception.^5,7^ Studies were limited by small sample size, little or no adjustment for confounders, and short follow-up which limits comparison with our study. Our results suggest no robust difference in glucose-related traits at any age up to young adulthood, which agrees with some but not all previous studies which were mostly done in children.^5–7^

While the mechanisms by which ART can lead to cardiometabolic differences are unknown and require investigation, one explanation for the different childhood to adulthood patterns in blood pressure, and possibly adverse TG trajectory with older age, is that they reflect age-specific differences in adiposity and their effect on cardiometabolic health. This is supported by findings from 26 ART-Health cohorts showing smaller size and lower adiposity during early life in ART-conceived than NC offspring and subsequently higher adiposity in young adulthood,^18^ a finding also reported in adults from Nordic registries.^44^ Higher adiposity increases blood pressure and TG^17,45^ and so age-related differences in adiposity could plausibly explain our findings. Differences in epigenetic and metabolomic profiles (possibly secondary to inherited causes of infertility, ART, or pregnancy-related complications) are other potential mechanisms.^14,46–48^

While higher blood pressure and TG are known to increase future cardiovascular disease risk^49–52^ and this might suggest an increased risk in ART-conceived offspring, the differences found in our study were small and mostly statistically non-significant. Therefore, our findings should be considered largely reassuring for ART users, although we cannot rule out later life problems. Similarly, while evidence suggests higher LDLc^52,53^ but not HDLc^52,54,55^ raises cardiovascular disease risk, the differences we found were small and did not persist to young adulthood.

Strengths of this study include the large sample size in comparison with previous studies and the inclusion of cohorts from different geographic regions, which should make the findings generalizable to ART pregnancies from various countries. The use of cohorts with comparison groups from the same underlying population as those conceived by ART is another important strength, which is not the case with many clinical cohorts where controls are selected from relatives or friends of the couples undergoing ART. Our novel trajectory analysis is of further strength as it allows both change with age and, importantly, a wider age range to be explored, thus providing insight into evolution of cardiometabolic risk factors over the life course.

Study limitations include the low precision/power (and relatively small number of ART conceptions) for some outcomes even with this large collaboration. Most cohorts were young which meant we were unable to examine results by age groups and could only do trajectory analyses in a subgroup of the included cohorts. Whilst our analyses were adjusted for pre-specified confounders, residual confounding by parental health conditions and other unmeasured factors is possible. Family designs such as within sibling and twins comparisons might have provided better control for confounding by family/genetic background; however, this design was not possible in our study because of the very large sample sizes that would be needed.^14^ Additionally, our subgroup analysis comparing ART conceptions with NC born to fertile and subfertile parents suggests that an underlying infertility, through which parental ill health would cause use of ART, is unlikely to explain our findings.

Our analysis of all cohorts was restricted to offspring with complete data on mode of conception, confounders, and outcomes which may have reduced precision of estimates and introduced a bias due to missing data. Exclusion of offspring with missing data on conception mode and confounders may also have reduced estimate precision from our trajectory models, although bias due to missing outcome data may have been reduced in our trajectory analyses by including all offspring with incomplete outcome data. Our analysis samples were more well off than those excluded due to missing data, which might limit generalizability of our findings to lower socioeconomic groups.

We explored if our findings could be due to multiple births by examining associations in singletons only and did not examine associations separately in multiple births because this would be underpowered, and likely to be less relevant to current and future generations given the declining prevalence of multiple pregnancy with ART.^56^ Mediation through offspring birth weight, gestational age, and BMI was examined by adjusting for these characteristics. These results should be interpreted with caution since potential violation of the assumptions for this approach to mediation analyses, and potential for collider bias, makes them difficult to interpret.^22,23,57^ We did not explore possible mediation by adolescent or adulthood BMI, which may be important for results at older ages, but if done within a multivariable framework it would need a cautious interpretation. Lastly, we were unable to investigate mediation by congenital heart disease^3,4^ due to its low prevalence across cohorts.^58^

## Conclusions

We found no adverse differences in HR or glucose-related traits between ART-conceived and NC children but found evidence of raised lipids in childhood that did not persist to young adulthood, and some evidence of more adverse blood pressure and TG trajectories to young adulthood in those conceived by ART. Overall, our findings should be deemed largely reassuring to people conceived by ART. Studies with longer follow-up are needed to examine associations of ART with cardiometabolic health across adulthood, and investigate mechanisms that might link ART to subsequent outcomes, if evidence does emerge in later adulthood. Future research on epigenetics, metabolomics, and cardiovascular and arterial phenotypes may provide insight into possible underlying mechanisms.

## Supplementary material

Supplementary material is available at *European Heart Journal* online.

## Supplementary Material

ehac726_Supplementary_Data

## Data Availability

The data used in this analysis are available to bona fide researchers by application to each of the participating cohorts, see Supplementary material online, *Text S2* for more details.
